# Supplementary material for: Evidence supported by Mendelian randomization: impact on inflammatory factors in knee osteoarthritis
Source: Front Med (Lausanne). 2024 May 28;11:1382836. doi: 10.3389/fmed.2024.1382836 (PMC11165061; doi:10.3389/fmed.2024.1382836)
Supplement: Supplementary file 1 [file Data_Sheet_1.ZIP › data/GCST90274776_5e6/776.forest.pdf]

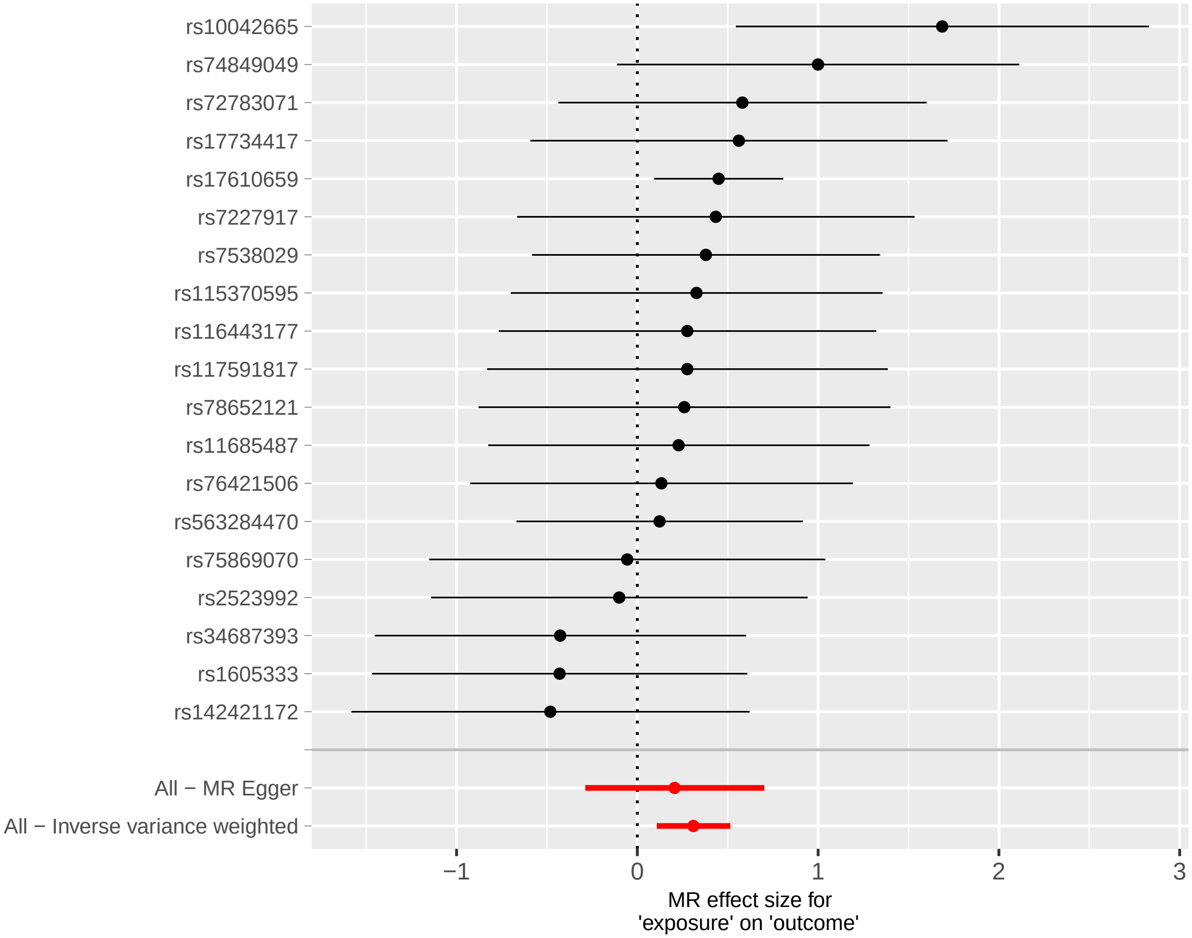

rs10042665
rs74849049
rs72783071
rs17734417
rs17610659
rs7227917
rs7538029
rs115370595
rs116443177
rs117591817
rs78652121
rs11685487
rs76421506
rs563284470
rs75869070
rs2523992
rs34687393
rs1605333
rs142421172
All − MR Egger
All − Inverse variance weighted
−1
0
1
2
3
MR effect size for
'exposure' on 'outcome'
